# Establishment and clinical validation of an in-cell-ELISA-based assay for the rapid quantification of Rabies lyssavirus neutralizing antibodies

**DOI:** 10.1371/journal.pntd.0010425

**Published:** 2022-05-10

**Authors:** Lara Schöler, Vu Thuy Khanh Le-Trilling, Ulf Dittmer, Melanie Fiedler, Mirko Trilling

**Affiliations:** Institute for Virology, University Hospital Essen, University of Duisburg-Essen, Essen, Germany; Minia University, EGYPT

## Abstract

Neutralizing antibodies (nAbs) prevent the entry of viruses into permissive cells. Since nAbs represent correlates of protection against the Rabies lyssavirus, the presence of sufficient nAbs indicates effective vaccination. Accordingly, Rabies lyssavirus-specific nAb titers need to be determined in routine diagnostics to identify individuals being at risk of Rabies lyssavirus infections due to insufficient immunity. The current gold standard for the quantification of Rabies lyssavirus-specific nAbs is the *rapid fluorescent focus inhibition test* (RFFIT). However, RFFITs are expensive and labor-intensive since multiple microplate wells must be evaluated one-by-one by trained personnel through microscopic inspection, which limits the number of samples that can be processed. To overcome this disadvantage, we established a novel assay for Rabies lyssavirus-specific nAbs relying on an in-cell-ELISA (icELISA)-based neutralization test (icNT). The icNT differs from the RFFIT in the readout phase, and can be automatically quantified in minutes using broadly available microplate readers. During the establishment, icNT parameters such as antibody concentrations, permeabilization procedures, blocking reagents, infectious doses, and the duration of infection were optimized. Afterwards, a dose-dependent detection of Rabies lyssavirus neutralization was demonstrated using the WHO Standard Rabies Immunoglobulin reference. A panel of 200 sera with known RFFIT titers revealed very good sensitivity and specificity of the icNT. Furthermore, the icNT showed very good intra- and inter-assay precision. By recognizing Rabies lyssavirus-specific antigens, the assay can be applied immediately to automatically quantify the concentration of Rabies lyssavirus nAbs in routine diagnostics or for various basic research questions such as screening for antiviral compounds.

## Author summary

Rabies is caused by the Rabies lyssavirus, for which no approved antiviral drug is available, and that is one of the deadliest virus infections that kills almost all individuals who develop symptoms. According to the World Health Organization, millions of disability-adjusted life years are lost each year, and approximately 59,000 people die from rabies. Since rabies can be prevented by a very safe and very effective vaccine, aforementioned loss is particularly tragic.

For residents of areas where the Rabies lyssavirus is endemic as well as travelers visiting these regions, vaccination and the verification of protective immunity conferred by neutralizing antibodies are crucial for the global fight against rabies. To optimize this process, we have developed and validated a simple, reliable, and cost-effective assay for the quantification of antibodies that neutralize the Rabies lyssavirus. The assay is based on an in-cell-ELISA approach. We are convinced that our method is suitable to replace the currently used expensive and labor-intensive methods, so that additional neutralization tests can be performed. This could help our colleagues in developing regions such as the tropics, which are disproportionately affected, in the fight against rabies.

## Introduction

Rabies is a ubiquitous zoonotic disease caused by the neurotropic Rabies virus (Rabies lyssavirus), which is a member of the genus Lyssaviruses, belonging to the family of *Rhabdoviridae* [1]. With a mortality rate of up to 100% [2], Rabies lyssavirus is among the infectious agents with the highest case-fatality rate. Dogs are the main carriers and the primary reservoir worldwide [3], but Rabies lyssavirus can also circulate in a variety of other mammalian, predominantly carnivorous, species [4]. For several recognized Lyssavirus species, bats are the principal reservoir [5]. In addition to carnivores, Rabies lyssavirus also circulates in New World chiroptera [6]. While the primary mode of transmission occurs through bites by infected animals, Rabies lyssavirus can also be transmitted in the context of transplantations and through contamination of mucous membranes and open wounds with virus-containing saliva and CNS fluids [7–13]. The probability of Rabies lyssavirus acquisition after a bite by an infected animal depends on factors such as viral loads in the saliva, the virus strain, and the site of the bite [14]. The usage of cellular entry receptors, e. g., *neuronal cell adhesion molecule* (NCAM) and *p75 neurotrophin receptor* (p75NTR) [15] together with several virulence factors (see e.g., [16,17]) enable Rabies lyssavirus to enter peripheral neurons and replicate there, followed by a retrograde transport to the central nervous system [17]. Although most rabies patients develop symptoms during a period of one to three months after infection, the local replication as well as the efficacy of the retrograde axonal transport to the central nervous system can influence the highly variable incubation time, which can range from weeks to years [14]. Rabies lyssavirus causes an acute encephalitis, manifesting in humans either as the more common furious or as the paralytic syndrome. Typical characteristics of the furious form are hydrophobia, aerophobia, and/or aggressive behavior, while flaccid paralysis is the typical manifestation of the paralytic form [14,18]. Unfortunately, once symptoms appear, the disease almost inevitably leads to coma and death—typically by respiratory or heart failure [2,18,19].

For some reasons, rabies appears to be frequently underestimated or even neglected [17,20]. It is known for approximately 4,000 years [21] and approximately three billion people live in or travel through endemic regions. According to the WHO, each year more than 59,000 people die as consequence of rabies diseases [22]. Since children under the age of 15 are often affected in Asia and Africa [18], Rabies lyssavirus infections annually cause a loss of over 3.7 million disability-adjusted life years (DALYs). Taken together, rabies constitutes a major public health concern affecting many parts of the global population [23,24]. The toll in morbidity and mortality is particularly upsetting given that rabies is preventable by a safe and effective prophylactic vaccine. Prior to symptom onset, the vaccine can even be applied as post-exposure prophylaxis [18,25]. In the absence of complete pre-exposure vaccination or if the individual is immuno-suppressed, the active immunization should be supplemented with the application of human Rabies immunoglobulin (HRIG) given as passive immunization [18,26]. In all cases and types of pre- or post-exposure vaccination, neutralizing antibodies (nAbs) are considered to fulfil the protective role [18,27]. The WHO recommends the vaccination for people at risk of exposure such as veterinarians, laboratory workers, and people who live in endemic areas or travel there. For people with prolonged risk of exposure, the WHO further recommends regular nAb titer controls [19,20,28]. If nAb titers fall below 0.5 IU/ml, a booster vaccination is recommended. Currently accepted methods to determine Rabies lyssavirus nAb titers are the above mentioned RFFIT [29] and the fluorescent antibody virus neutralization test (FAVN) [30]. With proper validation, WHO also allows classic ELISAs, recognizing binding antibodies, for the evaluation of Rabies lyssavirus-specific immune responses, but the gold standard to determine the vaccine response by evaluating Rabies lyssavirus-specific nAb titers are the *fluorescent antibody virus neutralization (FAVN)* and the *rapid fluorescent focus inhibition test* (RFFIT) [22,28]. The RFFIT is a cell-based neutralization test (NT) using replication competent Rabies lyssavirus strains such as the CVS-11 [28]. The evaluation is based on microscopic inspection of infected cell cultures and the quantification of Rabies lyssavirus-induced foci labelled by a fluorophore-coupled monoclonal antibody [22,29]. Like all clinical laboratory tests, the RFFIT must be performed by trained personnel. Numerous modifications exist, but according to the Office International des Epizooties (OIE) and the WHO, either 25–50 fields of vision at a x160-200 magnification, or 20 fields of vision at x100-200 magnification shall be evaluated. Taking into account typical dilution series, at least 50 and up to 100 fields must be evaluated by microscopic inspection and counting [22,31] excluding repetitive parallel determinations that would even further increase this number. Obviously, this is time-consuming and expensive. Alternatively, “the percentage of infected cells is estimated by the reader and a percentage of infected cells (from 0% to 100%) is attributed to each well”[22], which circumvents counting but may result in a lower degree of objectivity and reproducibility [22]. Hence, the assay is time-consuming, expensive, to certain degree subjective, and restricted to laboratories with sufficient skilled personnel. These disadvantages are limiting factors for a broader application of RFFITs in developing regions in which Rabies lyssavirus circulates. Laboratories in high-income regions are also affected by these limitations, since the hands-on-time is a critical determinant in cost-efficient routine diagnostics. Therefore, we aimed to establish an optimized NT for Rabies lyssavirus and finally developed an in-cell-ELISA (icELISA) that can be automatically quantified using broadly available microplate readers to rapidly provide quantitative information concerning the Rabies lyssavirus immunization status.

## Material and methods

### Sera, controls, cells, and virus

Clinical serum samples were obtained from the routine diagnostics and stored at -20°C. WHO-2 Standard Rabies Immunoglobulin (SRIG) (code RAI, NIBSC 30 IU) and HRIG (Berirab, CSL Behring GmbH, No: 107a/89) were adjusted to 1 IU/ml by dilution and served as references. Baby hamster kidney fibroblasts subclone 13 (BHK-21 C13; ATCC CCL-10) cells were grown in MEM Eagle with Earle’s Balanced Salt Solution (EBSS), L-Glutamine (292 mg/L), and 2.2 g/L NaHCO_3_ (PAN-Biotech Cat. No. P04-08500) supplemented with 10% (v/v) fetal calf serum (FCS), 1% (v/v) ZellShield (Minerva cat. no. 13–0150), and NaHCO_3_ (Merck cat. no. 1.06329.1000) solution to get a final pH of 7.5–7.8. Cells were cultivated at 37°C in an atmosphere of 5% CO_2_ and approved to be free of mycoplasma periodically. Challenge Virus Standard (CVS-11) stock was received from Friedrich-Löffler-Institut, Wusterhausen, Germany. Virus stocks were aliquoted and stored at -80°C. Viral titers were determined by TCID50 titration. All steps in which CVS-11 was handled prior to chemical fixation and virus inactivation were conducted in a BSL2 laboratory specifically dedicated to Rabies lyssavirus work. Only employees who had been successfully vaccinated against Rabies lyssavirus had access to this area.

### Ethics statement

The assessment of existing samples for the improvement of diagnostic procedures has been approved by the ethics committee of the Medical Faculty of the University of Duisburg-Essen (approval number #18-8309-BO). Residual material from clinically indicated tests was applied for the establishment and validation of the novel diagnostic procedure (icNT). For this purpose, donors remained anonymous and no personal data were included in the analysis. Therefore, individual consent was not obtained.

### Rapid fluorescent focus inhibition test RFFIT

The RFFIT was performed as version modified for 96-well microtiter plate platform format. Each test included WHO-2 SRIG, HRIG, and internal standards that were tested in the same manner as serum samples. Prior to the use, serum samples were heat-inactivated at 56°C for 30 minutes to destroy the complement. Five-fold serial dilutions of serum samples and controls were prepared with an initial predilution of ½ and a final volume of 100μl. 100μl Rabies lyssavirus (adjusted to 1000 TCID50 per ml; TCID50: median tissue culture infectious dose) was added and incubated at 37°C. After 70 minutes, 100μl freshly trypsinized BHK-21 cells were added (2.4x10^5^ cells/ml). At 22 hours post-infection, the supernatant was aspirated, cells were rinsed once with 300μl cold 80% (v/v) Acetone/PBS per well and fixed with cold 80% (v/v) Acetone/PBS for 10 minutes at -20°C. Following fixation, cells were rinsed two times with PBS. The Anti-Rabies Monoclonal Globulin (50μl/well) (Fuji Rebio Cat# 800–092, RRID:AB_2802166) was added at a 1/200 dilution and incubated for 35–40 minutes at 37°C. Following incubation, cells were rinsed two times with PBS. Rabies lyssavirus-infected cell foci were manually counted by fluorescence microscope. Six fields of vision were examined per well at x400 magnification to score foci. The Spearman-Karber method was used to calculate 50% endpoint (ED_50_). The ED_50_ titer of the test serum was then transformed into international units (IU)/ml by comparing the sample titer with the WHO-2 SRIG titer with a known potency value.

### In-cell-ELISA-based neutralization test (icNT) for Rabies lyssavirus

The icNT was performed on 96-well plates while each test included WHO-2 SRIG, HRIG, and internal standards, tested in the same manner as serum samples. Prior to use, serum samples were heat-inactivated at 56°C for 30 minutes to destroy the complement. A detailed icNT protocol is provided in the S1 Text. Briefly, 2.5-fold serial dilutions of serum samples and controls were prepared with an initial predilution of 1/10 and a final volume of 100μl. Rabies lyssavirus (4 PFU/cell) was added and incubated. After 70 minutes, 50μl freshly trypsinized BHK-21 cells were added. The cell number was adjusted in order to reach a confluent cell layer with approximately 5x10^4^ cells/well of a 96-well plate at the time point of analysis. At 48 h post-infection, cells were fixed for 15 minutes in 3.5% (w/v) paraformaldehyde/PBS, by addition of 150μl/well 8% (w/v) PFA, followed by three washes with PBS. By adding 100μl PBS, plates can be stored for several days at 4°C. Cells were permeabilized with 1% (v/v) Triton-X-100/PBS for 30 minutes and blocked with 3% (v/v) FCS/PBS for two hours. The Anti-Rabies Monoclonal Globulin (50μl/well) (Fuji Rebio Cat# 800–092, RRID:AB_2802166) was added at a 1/4200 dilution and incubated overnight at 4°C or for 35–40 minutes at 37°C. Following incubation, cells were washed three times with 0.05% (v/v) Tween-20/PBS and peroxidase-labelled goat anti-mouse antibody (Jackson ImmunoResearch Labs cat. no. 115-035-003, RRID:AB_10015289) at 1/2000 dilution was added, incubated for two hours and washed four times with 0.05% (v/v) Tween-20/PBS. Tetramethylbenzidin (TMB) substrate was added and the ELISA reaction was started. The reaction was stopped with 0.5 M HCl. Subsequently, the absorbance was measured using a microplate multireader (Mithras^2^ LB 943; Berthold Technologies).

### Statistical analysis

Statistical analysis was performed with GraphPad Prism (Version 6.0). Results were expressed as mean plus/minus standard deviation (SD). Significance was calculated by an unpaired two tailed t test. Results with p values less than 0.05 were considered significant. The coefficient of determination was used to analyze the assay linearity. The Pearson correlation coefficient was used to analyze the linear regression. The coefficient of variation (CV) was determined [%CV = (SD (standard deviation)/average) x 100] to analyze intra- and interassay precision. The calculation of sensitivity and specificity was done using algorithms online available at https://www.medcalc.org/calc/diagnostic_test.php.

## Results

**Fig 1:**
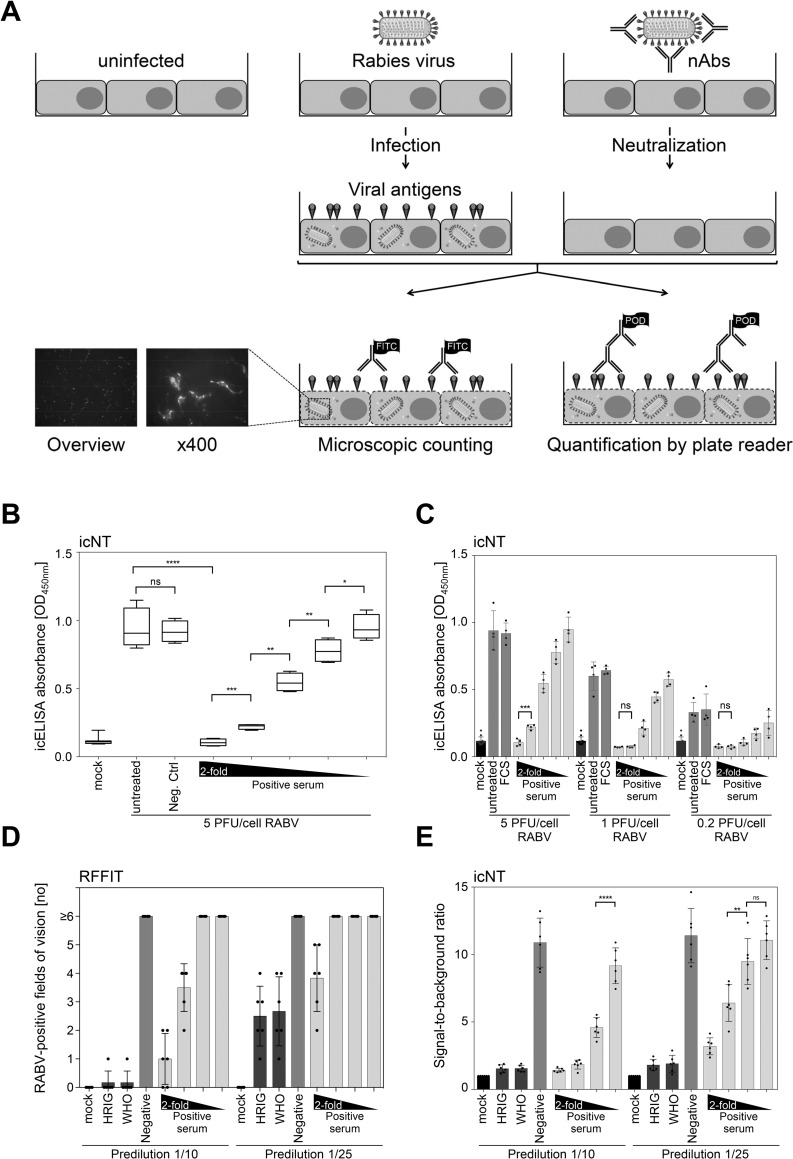
The icNT results correlate with the RFFIT. (A) Schematic overview of the newly established assay, icNT, and the currently used assay, RFFIT. Serum samples were incubated with Rabies lyssavirus. After 70 minutes, a suspension of BHK-21 cells was added. For the analysis by icNT at 48 hours post-infection (h p. i.), cells were permeabilized and infected cells were detected by a specific primary antibody. A peroxidase-labelled secondary goat anti-mouse antibody was added and the ELISA reaction was started by use of TMB substrate and stopped with 0.5 M HCl. The absorbance was quantified by use of a microplate multireader. For the analysis by RFFIT at 22 h p. i., infected cells were detected by a specific FITC-labelled primary antibody and evaluated microscopically. (B) Rabies lyssavirus was incubated with a negative control serum, a positive control serum or left untreated. A twofold dilution was done with the positive control. Neutralization was evaluated by icNT. 4-fold replicates of samples were determined. The boxes range from the 25^th^ to the 75^th^ percentile. The line within the box depicts the median value. The whiskers indicate minimum and maximum values. Significance was calculated by an unpaired two-tailed t test, *p < 0.05; **p < 0.01; ***p < 0.001; ****p < 0.0001. Linear regression for the linearity was analyzed by coefficient of determination, R^2^ = 0.94. (C) icNT was performed with different doses of infection of Rabies lyssavirus (MOI 5, 1 and 0.2). (D)(E) RFFIT and icNT were performed in parallel. Same sera and same dilutions were used. Bars depict the mean values ± SD. Dots show the values of the individual measurements. Samples were analyzed in sixfold determinations.

To overcome aforementioned limitations of RFFIT, we set out to establish and validate an icELISA-based Rabies lyssavirus neutralization test (icNT) applying ELISA microplate readers, which are usually present at routine diagnostics laboratories, to automatically quantify the Rabies lyssavirus infection and the inhibition by nAbs. **Fig 1A** displays a schematic comparison of the RFFIT and the icNT. It is worth mentioning that the Rabies lyssavirus-permissive cells, the replicating Rabies lyssavirus strain, and the Rabies lyssavirus-specific antibody applied in the WHO-approved RFFIT and in the herein described icNT are all the same. Thus, the principle mode of recognition is virtually identical.

### Establishment and optimization of the icELISA

Based on our aim to set up an assay that provides precise information on the Rabies lyssavirus-specific nAb status and enables automated readout, an icELISA-based approach for the recognition of Rabies lyssavirus antigens was established. A direct transfer of all experimental RFFIT parameters concerning the infection conditions including the low MOI into the icELISA did not yielded sufficient signal to noise ratios. Therefore, all relevant experimental parameters were carefully evaluated and optimized to improve the signal-to-background ratio. In the end, we obtained optimal results using the following conditions: two days of Rabies lyssavirus infection (S1 Fig) similar to FAVN [30], a fixation of cells by using a final concentration of ~3.5% (w/v) PFA (S2 Fig), permeabilization of cells with 1% (v/v) Triton-X-100 (S3 Fig), a blocking of unspecific background using 3% (v/v) FCS (S4 Fig), and a primary antibody dilution between 1/3200 and 1/6400 (S5 Fig). Without a permeabilization step or when other detergents were used, the icELISA did not provide sufficient signal-to-background signals (S3 Fig), suggesting that the response largely relies on intracellular viral antigens. Conversely, different blocking reagents can be applied (S4 Fig). Please note that the icELISA signal was largely diminished if infected cells were not incubated long enough to enable Rabies lyssavirus replication (S1 Fig) or not permeabilized appropriately (S3 Fig), indicating that intracellular Rabies lyssavirus antigens generated during Rabies lyssavirus replication dominate the icELISA signal.

### The icELISA allows a quantitative assessment of Rabies lyssavirus antigens

After the optimized icELISA approach for Rabies lyssavirus had been established, we tested its applicability for neutralization tests. A Rabies lyssavirus-positive control serum sample with a known high titer of Rabies lyssavirus-specific nAbs was compared to a negative control serum sample, both of which used as standard controls in the routine diagnostics. As expected, Rabies lyssavirus-infected cells induced a pronounced icELISA signal in comparison to the mock-infected control cells. While the non-neutralizing serum sample did not reduce the signal, the serum sample with Rabies lyssavirus-specific nAbs reduced the icELISA signal-to-baseline ratio, indicating that an icELISA-based icNT can be applied to identify sera with Rabies lyssavirus-neutralizing capacities (**Fig 1B**). To test if the icNT is capable to report on quantitative differences in terms of the quantity of Rabies lyssavirus-specific nAbs, serial two-fold dilutions of the serum sample containing Rabies lyssavirus-specific nAbs were assessed. We observed a stepwise and significant increase in the Rabies lyssavirus-specific signal upon dilution of the nAbs (**Fig 1B**), indicating that the icNT is applicable to discriminate between different levels of Rabies lyssavirus neutralization.

### The neutralization of Rabies lyssavirus dose-dependently diminishes the icELISA signal

To test whether there is a correlation between the icELISA signal, the Rabies lyssavirus infectious dose, and the neutralization, three different infectious doses (5, 1, and 0.2 PFU/cell) were comparatively assessed. As expected, the Rabies lyssavirus-specific icELISA signal correlated very well with the infectious doses (**Fig 1C**). In all cases, serum samples containing Rabies lyssavirus-specific nAbs dose-dependently reduced the icELISA signal (**Fig 1C**). Please note that only the highest serum concentration fully neutralized the high MOI infection (5 PFU/cell), whereas infections performed with lower MOIs (1 or 0.2 PFU/cell) were fully neutralized by more diluted serum. This indicates that the icNT is capable to discriminate gradual differences in terms of the relationship between virus loads and Rabies lyssavirus-specific nAbs.

### RFFIT and icNT show comparable results

To validate the icNT for possible use in routine diagnostics, RFFIT and icNT were performed side-by-side. The same serum samples and controls as well as same dilutions were applied to both tests in parallel. As expected, an inverse correlation between RFFIT and icNT responses and the concentration of Rabies lyssavirus-specific nAbs was evident. The comparison indicated that RFFIT and icNT in principle yield similar results (**Fig 1D and 1E**). However, due to the underlying test principle, the RFFIT did not resolve differences of the higher dilutions, since the evaluation is based on a binary decision system in a way that evaluated fields of vision either contain infected cells or not. One field of vision is judged as positive irrespective of the actual number of infected cells present in this field of vision. Conversely, the icELISA generates continuous data sets proportional to the amount of antigen and the number of infected cells (**Fig 1E**). In our hands, the icNT was superior to the RFFIT in terms of data quality and data granularity—at least in this RFFID modification and pre-dilution schema and when a limited number of serum dilutions that is feasible for routine diagnostics are assessed.

**Fig 2:**
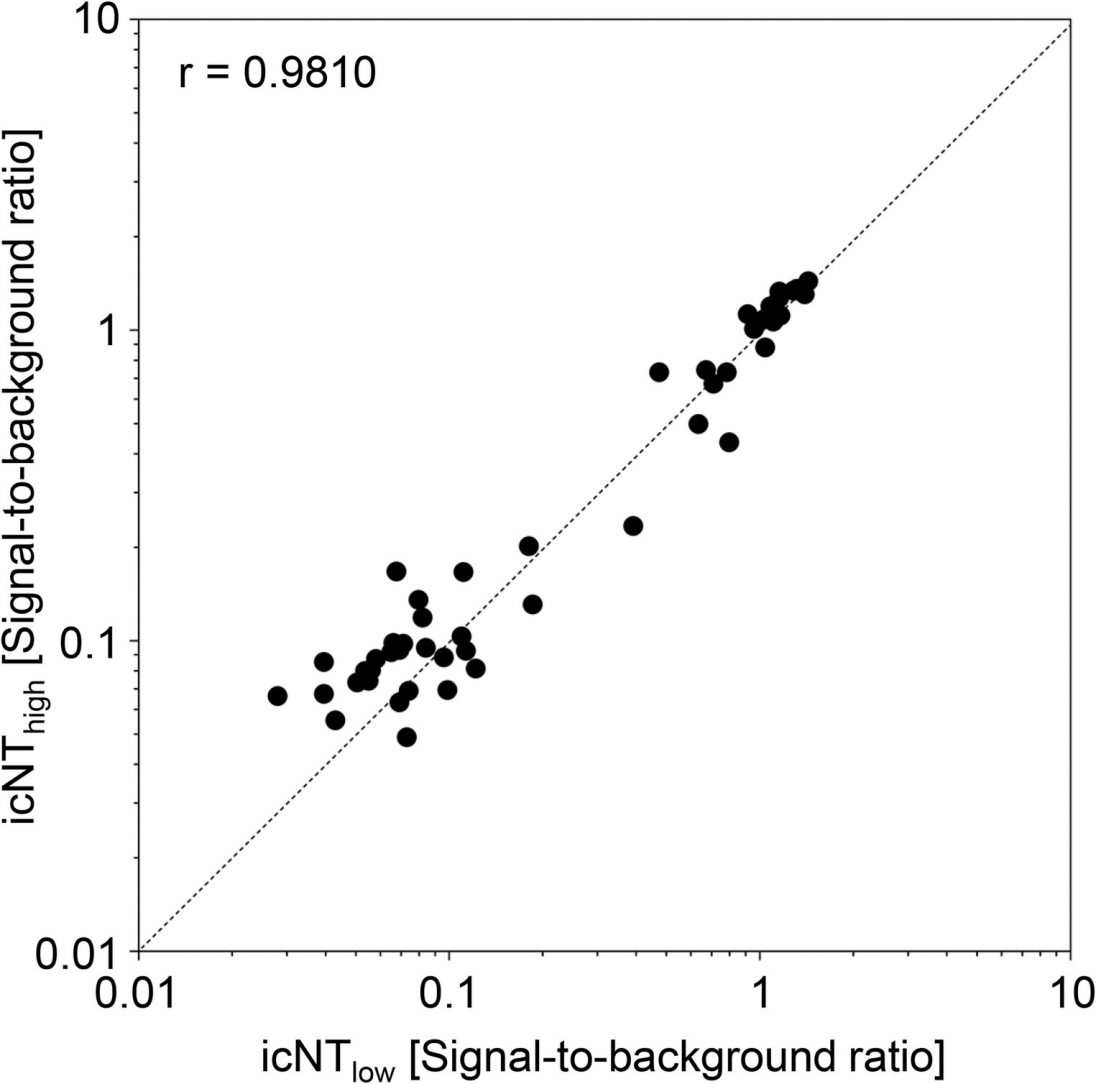
The increased virus inoculum does not affect icNT results. Two icNT protocols were performed in parallel either using the usual “high” dose of Rabies lyssavirus (see standard protocol) or a “RFFIT-like” low-dose infection (~100 TCID50 per well). The high-dose version was fixed, processed, and evaluated by icELISA at 48 h p. i., while the low dose icNT was analyzed at 72 h p. i. to allow replication of the non-neutralized residual virus. Samples were determined in duplicate.

In comparison to the classical RFFIT, our icNT protocol applies higher virus doses. Since this difference may affect the outcome, we sought to test the influence of this alteration. However, the RFFIT is not easily applicable with increased virus titers because it would result in more foci than can be differentiated and counted. The icNT in turn requires sufficient viral antigens for an optimal signal-to-background ratio. To address whether the increased virus inoculum used in our icNT affects the neutralization, we performed an icNT using a low virus dose that is applied in RFFIT assays (1000 PFU per well), establishing “RFFIT-like” conditions during the incubation period during which antibodies recognize and neutralize the virions. To compensate for the low amounts of viral antigens, we afterwards allowed the non-neutralized virus to replicate for 72h. This “RFFIT-like” icNT and our usual icNT protocol yielded almost identical results (**Fig 2**), indicating that increased virus doses do not significantly influence the test results.

### The icNT shows very good intra- and interassay precision

**Fig 3:**
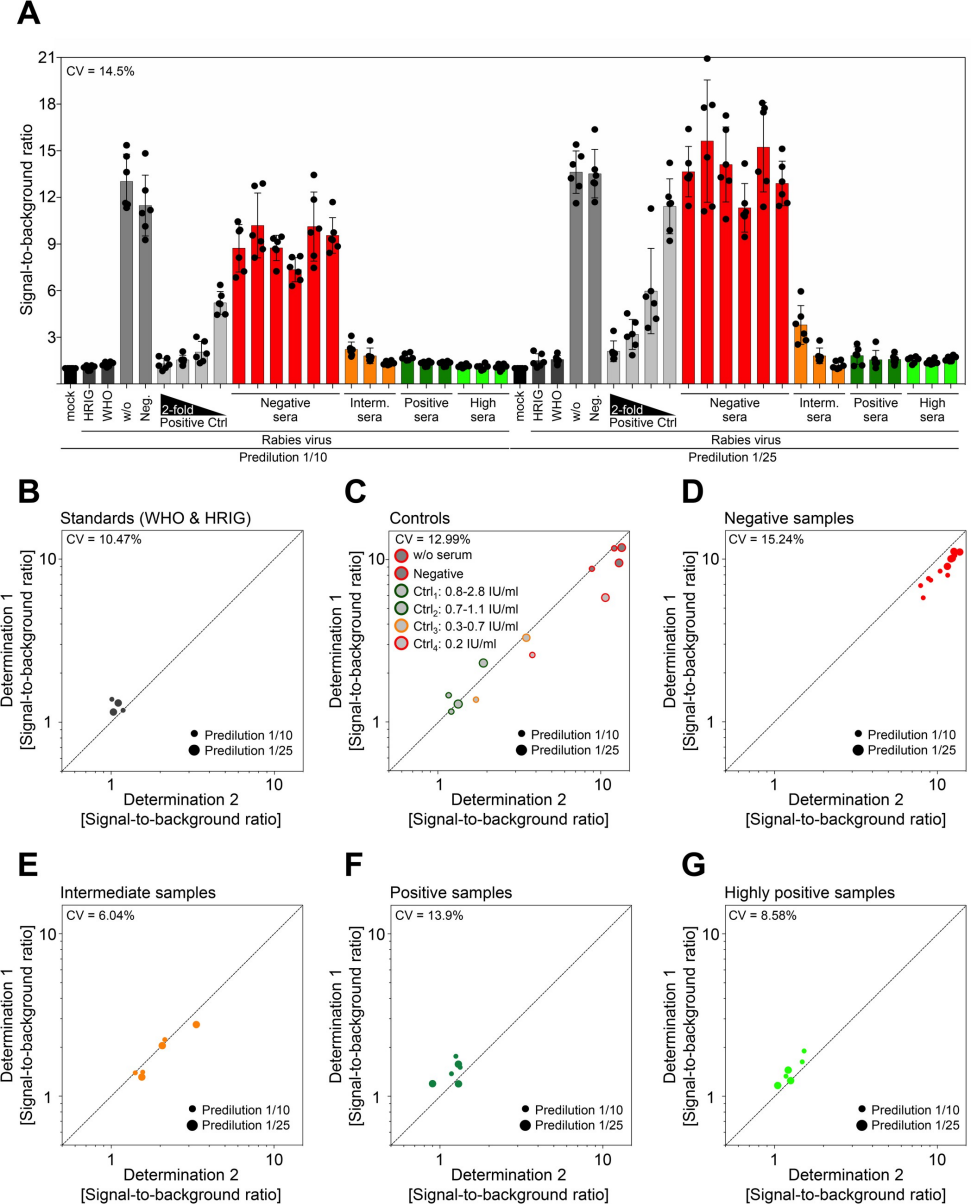
The icNT shows very good intra- and interassay precision. (A) HRIG, WHO-2 SRIG, negative control, twofold dilution of positive control, six negative, three intermediate, three positive, and 3 highly positive serum samples with known RFFIT titers were analyzed by icNT. 6-fold replicates of samples within one microplate were determined (intraassay precision). Bars depict the mean values ± SD. Dots show the values of the individual measurements. (B)-(G) Same samples as in (A) were determined on two different days (interassay precision). The abscissa and the ordinate each represent one determination. The dashed line represents a Coefficient of Variability (CV) of 0%. Dots show the values of the individual measurements. The determinations were grouped and visualized in separate graphs. Linear regression for the interassay precision was analyzed by the Pearson correlation coefficient, r = 0.98, p (two-tailed) = 0.0001. Samples were determined in duplicate.

In order to validate a novel diagnostic test for clinical routine application, sufficient intra- and interassay precision shall be confirmed. To evaluate potential variations within icNT results, multiple replicates of samples were measured on the same 96-well plate. For this purpose, a six-fold determination of every sample was conducted in one run (intraassay precision). Based on the primary data, it was immediately evident that the six replicates cluster together very well (**Fig 3A**). To further evaluate the reproducibility, the coefficients of variation (CV) was calculated.

The icNT results had a low overall CV of 14.5% indicating good reproducibility. Furthermore, the same samples were determined in independent assays at two different occasions to assess the interassay precision. For comparison, the Pearson’s correlation coefficient was calculated (**Fig 3B, 3C, 3D, 3E, 3F and 3G**), yielding in a very good coefficient value (r = 0.98; including all data shown in B-G), confirming a high interassay precision. Furthermore, CVs for stratified neutralization subgroups such as negative and positive samples were calculated. For each category, a low CV was apparent, emphasizing the very good interassay precision for the entire set of standards, controls, negative, intermediate, and positive samples. Overall, the icNT data had a CV of 11.2% (**Fig 3B, 3C, 3D, 3E, 3F and 3G**), indicating a high degree of interassay reproducibility enabling the application of the Rabies lyssavirus icNT in clinical routine diagnostics.

### The icNT shows very good sensitivity and specificity

**Fig 4:**
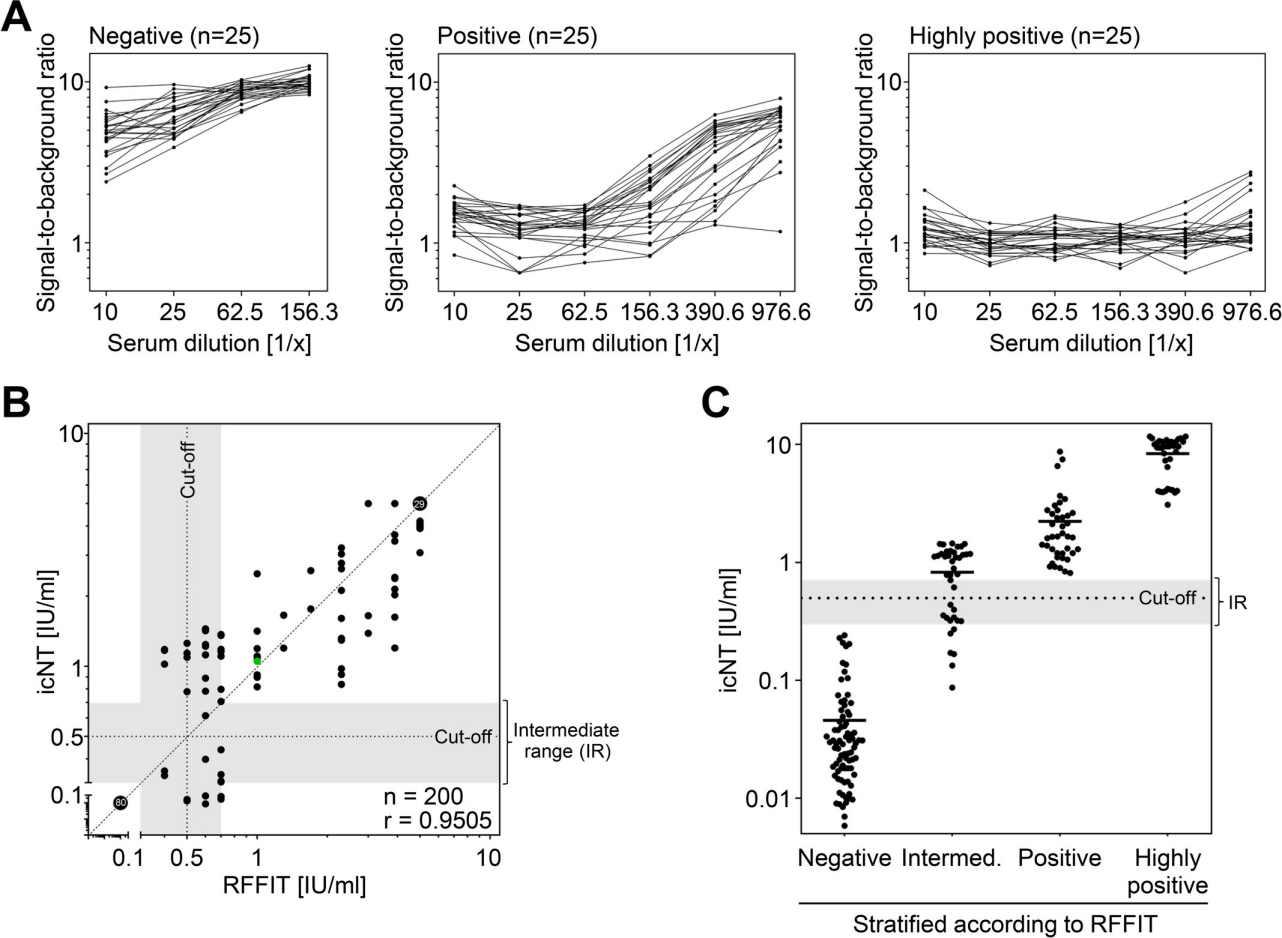
The icNT shows very good sensitivity and specificity. (A) 25 seronegative, 25 positive, and 25 highly positive exemplarily chosen serum samples with known RFFIT titers were analyzed by icNT at indicated serum dilutions. (B) Concordance of icNT versus RFFIT according to nAb titer, both calculated by the Reed-Muench method for n = 200 sera. Since our diagnostic RFFIT does not further stratify negative (<0.3 IU/ml) and highly positive (>4 IU/ml) samples, negative and highly positive sera were arbitrarily set to 0.1 and 5 IU/ml, respectively, in both assays. Please note: 80 negative and 29 highly positive sera are superimposed (indicated by bigger dots). The dashed line depicts the ideal linear regression line. The dotted line depicts the cut-off titer of 0.5 IU/ml and the intermediate range (0.3–0.7 IU/ml) is shaded in grey. Linear regression was analyzed by the Pearson correlation coefficient, r = 0.9505, p (two-tailed) < 0.0001. (C) icNT results of the sera shown in (B). Serum samples were stratified based on the RFFIT results to the indicated groups.

To evaluate the sensitivity and specificity, samples were comparatively analyzed by icNT and RFFIT. In the first approach, 50 serum samples were prediluted 1/10 and 1/25 and measured in both assays (S6 Fig). This dilution already enabled a distinction between Rabies lyssavirus neutralizing and non-neutralizing serum samples. To further increase the resolution of the icNT assay and to evaluate it, we exemplarily assessed 25 negative, 25 positive, and 25 highly positive sera at graded 2.5-fold dilutions ranging from 1/10 to 1/976.5625 (**Fig 4A**). Again, the icNT clearly distinguished between positive and negative sera. Furthermore, icNT was able to discriminate between positive and highly positive neutralizers if higher serum dilutions are taken into account (**Fig 4A**, right panel). To directly compare icNT results with reference results determined by RFFIT, the icELISA signal had to be converted into international units per ml (IU/ml). According to the WHO Rabies lyssavirus analysis guidelines, nAb titers have to be calculated by the Reed-Muench method, used to determine the 50% end point (ED_50_) titer of neutralization [22,32]. Subsequently, ED_50_ titers can be converted to IU/ml values by comparison to a defined reference serum sample such as the WHO Standard Rabies immunoglobulin [33]. According to the standard protocol, the determination of RFFIT ED_50_ titer is based on the evaluation of 80 fields (four dilutions and 20 fields of vision each) of vision per serum sample or the estimation of the percentage of infected cells by specifically trained personnel [22]. Based on the Reed-Muench method, the icELISA signal was converted into a percentage of infected cells, taking into account the axiom that the signal of the mock-infected control cells equals to zero percent infection and wells in which the virus was treated with a negative control serum, resulting in maximal infection, corresponding to 100 percent infection. Within these boundaries, the icELISA signal was calculated into a percentage of infection. The WHO defines a cut-off titer of 0.5 IU/ml as being the minimum protective antibody level. In the present analysis, this titer was used as discriminating titer based on which the results were stratified into positive and negative samples and then compared to RFFIT results. Based on the analysis of 200 serum samples (**Fig 4B**), 82 samples were negative in both assays and 103 were positive in both assays. The icNT indicated that 12 samples are negative despite RFFIT values exceeding 0.5 IU/ml. Three samples were negative in the RFFIT but positive in the icNT. Judged on these RFFIT data, the sensitivity and specificity of the icNT were 96.47% and 89.57%, respectively, and positive and negative predictive values were 87.23 and 97.17%, respectively.

As expected, some samples fall into the indiscriminate range of 0.3–0.7 IU/ml at which a clear interpretation is difficult (shaded in grey in **Fig 4B and 4C**). It is questionable, if different assessments would reliably come to the same conclusion for such border-line serum samples and the clinical decision would be to prescribe a booster vaccination in case of doubts of protection. When we stratified the results of the sera according to the RFFIT results into negative (<0.3 IU/ml), intermediate (0.3–0.7 IU/ml), positive (>0.7 and <4 IU/ml), and highly positive (>4 IU/ml) NT titers, and plotted the icNT results, it became apparent that aforementioned non-uniform results originate from the group of intermediate samples (**Fig 4C**). The accuracy of icNT was further tested by calculating the Pearson’s correlation coefficient. The overall accordance of RFFIT and icNT results based on a coefficient value of r = 0.9505 with a linear regression function of Y = 0.8984*X + 0.09491 (mean, median and maximal deviation from the regression function were ~0.155, ~0.085, and ~0.413, respectively). Based on the fact that the mean, median, and maximal deviation between RFFIT and icNT were 0.319, 0, and 2.701, respectively, the overall correlation between both assays was very good, emphasizing the high degree of consistency between RFFIT and icNT results.

## Discussion

The RFFIT has been the assay of choice for almost 50 years [29]. However, several authors emphasized important disadvantages of the RFFIT such as the intense hands-on-time, high costs, and the restricted degree of objectivity [34–37]. Moreover, WHO recommendations highlight the complexity of virus neutralization tests principles and point out that sensitivity and specificity depend on various factors. For optimal performance, stringent quality control procedures and specifically trained personnel are required [20]. Accordingly, others proposed imaging-based optimizations [34,38] that, however, have not gained broad application in the field. With the purpose of maintaining the advantages of the RFFIT and overcoming above-mentioned disadvantages, we established an icELISA-based Rabies lyssavirus neutralization test. The icNT assay is simple, rapid regarding the hands-on-time during the readout, objective, and a large number of samples can be evaluated simultaneously. Approximately 6–8 plates can be easily evaluated in one run, which is equivalent to processing 114–152 samples simultaneously. For this number of samples, the icNT requires an additional hands-on-time of about 1.5 hours for the pipetting steps. In contrast, performing the RFFIT would, according to the standard recommendation by the WHO, require microscopic readout of 9,120–12,160 fields of vision for the same number of samples, which is of course much more time-consuming than 1.5 hours and takes about one working day. In Germany, this additional working day would cost approximately 150€. Thus, per sample 1€ is saved. Furthermore, a lower dilution of the primary antibody is applied in the classical RFFIT (1/200 [v/v]) compared to the icELISA (1/4200 [v/v]), saving reagents and further reducing costs.

For ELISAs, acceptable CVs are below 20 [39]. It is well known, that cell-based neutralization assays exhibit a higher degree of variability. The values for intra- and interassay precision for the icNT were found to be 14.5% and 11.2%, respectively. Furthermore, all negative, positive, and highly positive serum samples were correctly diagnosed. Accordingly, the icNT had a sensitivity and specificity of 96.47% (95% confidence interval [CI]: 90.03–99.27%)% and 89.57% (95% CI: 82.48–94.49%), respectively, in an analysis of 200 sera, and correlated very well with the RFFIT (r = 0.9505; p<0.0001). All non-uniform result differing between RFFIT and icNT originate from samples that had shown an intermediate NT response in the RFFIT (0.3–0.7 IU/ml; **Fig 4C**). Those very good validation values confirm the high quality of icNT as well as the potency for its immediate application in routine diagnostics.

Compared to the RFFIT, three working steps have been added: cell permeabilization, incubation with an HRP-coupled secondary antibody, and the enzymatic TMB reaction. Despite these additional steps, the over-all hands-on time is still decreased, since the evaluation of the RFFIT is based on a time-consuming readout of 80 fields of vision per sample or alternatively an inaccurate rough estimate of the entire well [22]. To put it in a nutshell, the icNT needs a little bit more pipetting but far less microscopic counting efforts. One could argue that a disadvantage of the icNT is the dependence on a microplate/ELISA reader. However, such devices are present in almost all routine diagnostic laboratories. While ELISA readers are required for various routine diagnostic applications, fluorescent microscopes are often exclusively maintained and serviced in virology diagnostics laboratories for Rabies lyssavirus-specific nAb titer determinations.

Alternative neutralization tests have been established for Rabies lyssavirus, based on genetically modified viruses (e.g., GFP-expressing Rabies lyssavirus mutants [40]) or the application of genetically modified pseudotyped viruses [41]. Given that genetically modified viruses are usually either completely forbidden in routine diagnostics or at least associated with extensive bureaucratic and biosafety efforts, such approaches may be valuable for research but are not favorable for routine diagnostic purposes.

In the past, surrogate ELISA approaches have been proposed [35–37,42] that rely on the recognition of antibodies binding to Rabies lyssavirus-encoded proteins. Without measuring nAbs directly, such assays try to infer the vaccination status in terms of nAbs based on correlations between binding and neutralizing IgG. It may well be that the likelihood to have nAbs is to a certain extent correlated with the overall abundance of Rabies lyssavirus-binding antibodies. However, as in the case of most viruses, there is an intermediate range of uncertainty in which the level of binding antibodies does not faithfully discriminate between individuals with sufficient versus insufficient neutralization capacities. Mostly the envelope glycoprotein G is used as antigen source, since it is also the target of nAbs [37,43]. Obviously, the vast majority of antigen-binding antibodies recognizing a viral entry protein such as gG fail to elicit neutralizing capacities. Previous work further depicted that up to 1,000 monoclonal antibodies, directed to glycoprotein G, can bind a single Rabies lyssavirus virion without neutralizing it [27]. Hence, conventional ELISAs are simply inadequate as work-around for true neutralization tests, since they can only correctly predict protection for a fraction of sera. In contrast, our icNT detects genuine nAbs, which is paramount based on the fact that the presence of nAbs is essential to prevent Rabies lyssavirus infections [27,44]. In addition, conventional ELISA systems are vulnerable towards cross-reactions with antibodies against related viruses, whereas icNT is not affected by such inaccuracy, based on the use of one defined virus purposely introduced into the cell culture. In particular, for a disease with such a high case-fatality rate [20], an accurate and specific indication of the level of protection is of highest priority. Considering that successful vaccination is the sole means to prevent rabies [19], the vaccination status is of vital importance. Accordingly, none of the ELISA surrogate assays has replaced the RFFIT.

Moreover, others and we demonstrated that icELISA-based assays can quantify different viral antigens, e.g. derived from Cytomegaloviruses [45] and/or can be utilized to determine nAb titers of RNA viruses such as SARS-CoV-2 [46,47]. Given that we provide a third example here, it is tempting to speculate that icELISAs and icNTs can also be applied to other viruses for which the current methodology for the detection and quantification of nAbs is suboptimal.

Taken together, we established and clinically validated a rapid and cost-effective readout for Rabies lyssavirus-specific nAbs which constantly need to be quantified in routine diagnostics. We hope that others will make use of the provided laboratory protocol to independently evaluate, and ideally implement this assay.

## Supporting information

S1 Fig The icELISA signal can be largely enhanced by infecting cells for two days. BHK-21 cells were infected with Rabies lyssavirus using the indicated virus dose. At 9, 22, 29, 49, 55, 71 and 77 h p. i., cells were fixed and analyzed by icELISA. Bars depict the mean values ± SD. Dots show the values of the individual measurements. Four-fold replicates of samples were determined.
(TIF)

S2 Fig The background signal can be reduced by fixing cells with PFA. Rabies lyssavirus was treated with HRIG, WHO-2 (diluted in cell culture media), SRIG, WHO-2 aqua (diluted in water) without serum, negative control serum or positive control serum. A twofold dilution was done with the positive control. Sera were prediluted 1/10 or 1/25. At 48 h p. i., cells were fixed with 4% paraformaldehyde (PFA), 3.5% PFA or 80% acetone and analyzed by icELISA. Bars depict the mean values ± SD. Dots show the values of the individual measurements. Two-fold replicates of samples were determined.
(TIF)

S3 Fig Intracellular Rabies lyssavirus antigens generated during Rabies lyssavirus replication dominate the icELISA signal. BHK-21 cells were infected with Rabies lyssavirus using the indicated virus dose. At 22 h p. i., cells were fixed, permeabilized with 1% Triton-X-100, 0.2% Saponin, or without detergents and analyzed by icELISA. Bars depict the mean values ± SD. Dots show the values of the individual measurements. Cell were fixed after 22 h p. i. because the experiment was conducted prior to the experiments that assessed the influence of different infections periods. Four-fold replicates of samples were determined.
(TIF)

S4 Fig The background signal can be reduced by blocking with 3% FCS. BHK-21 cells were infected with Rabies lyssavirus using the indicated virus dose. At 22 h p. i., cells were fixed, blocked with 1% Bovine serum albumin (BSA), 5% milk powder (MP), or 3% fetal calf serum (FCS) and analyzed by icELISA. Bars depict the mean values ± SD. Dots show the values of the individual measurements. Cell were fixed after 22 h p. i. because the experiment was conducted prior to the experiments that assessed the influence of different infections periods. Four-fold replicates of samples were determined.
(TIF)

S5 Fig Prolonged infection time reduces the necessary amount of primary antibodies. BHK-21 cells were infected with Rabies lyssavirus using the indicated virus dose. (**A**) At 22 h p. i., cells were fixed and analyzed by icELISA using indicated dilutions of Anti-Rabies Monoclonal Globulin. Three-fold replicates of samples were determined. (**B**) At 48 h p. i., cells were fixed and analyzed by icELISA using indicated dilutions of Anti-Rabies Monoclonal Globulin. Four-fold replicates of samples were determined. Bars depict the mean values ± SD. Dots show the values of the individual measurements.
(TIF)

S6 Fig The icNT shows very good sensitivity and specificity. HRIG, WHO SRIG, negative control serum, twofold dilution of positive control serum, 20 seronegative, 10 intermediate, 10 positive, and 10 strongly positive serum samples with known RFFIT titers were analyzed by icNT. Serum samples were prediluted 1/10 or 1/25. The different graphs each represent one predilution. Each measurement was performed in duplicate.
(TIF)

S1 Text Supplementary information: icNT and icELISA protocol. A detailed laboratory protocol for the icNT and the icELISA is provided.
(DOCX)
