# Supplementary figures and images for: Establishment and clinical validation of an in-cell-ELISA-based assay for the rapid quantification of Rabies lyssavirus neutralizing antibodies
Source: PLoS Negl Trop Dis. 2022 May 10;16(5):e0010425. doi: 10.1371/journal.pntd.0010425 (PMC9159627; doi:10.1371/journal.pntd.0010425)

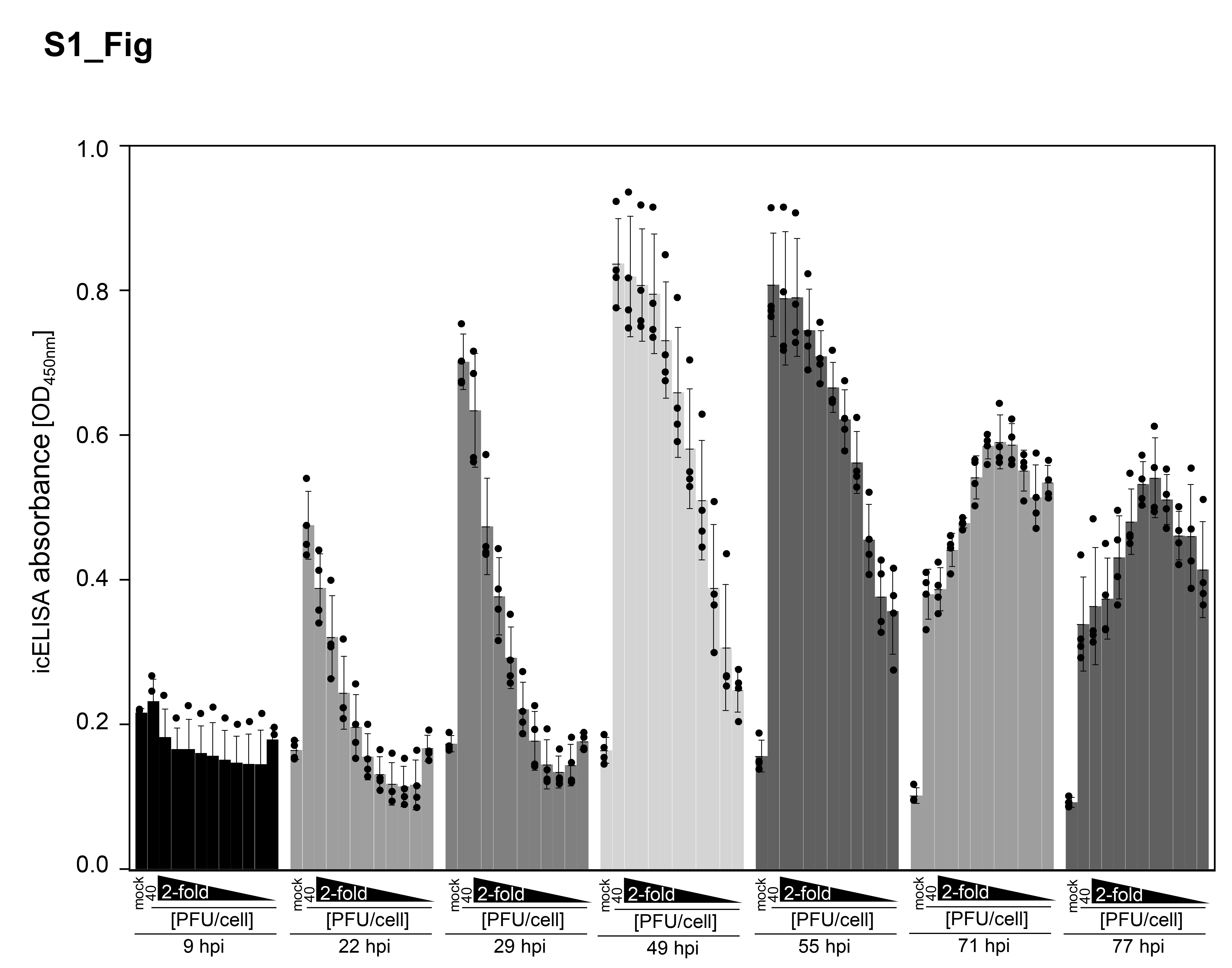

Supplement: S1 Fig — BHK-21 cells were infected with Rabies lyssavirus using the indicated virus dose. At 9, 22, 29, 49, 55, 71 and 77 h p. i., cells were fixed and analyzed by icELISA. Bars depict the mean values ± SD. Dots show the values of the individual measurements. Four-fold replicates of samples were determined. (TIF) [file pntd.0010425.s001.tif]

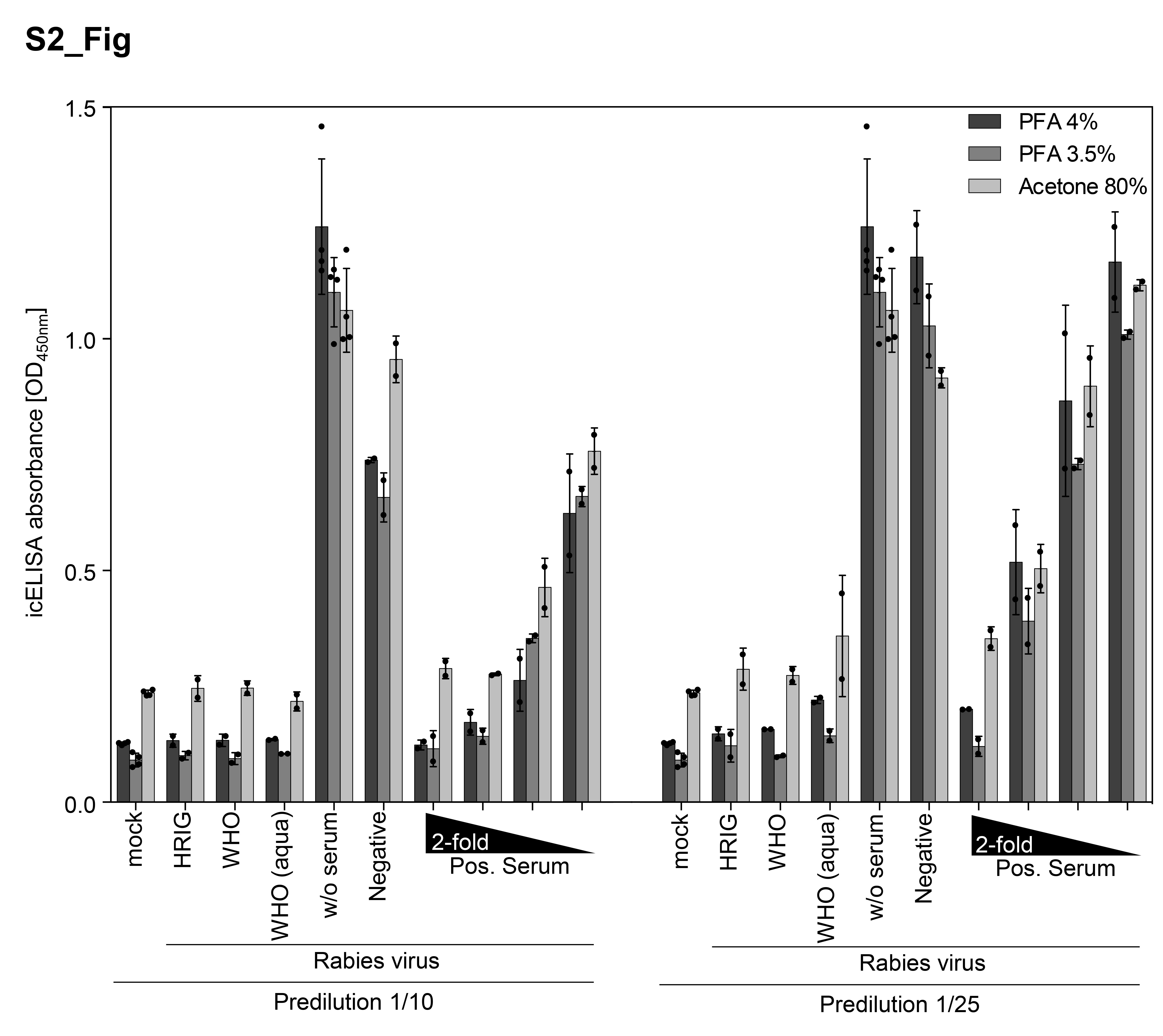

Supplement: S2 Fig — Rabies lyssavirus was treated with HRIG, WHO-2 (diluted in cell culture media), SRIG, WHO-2 aqua (diluted in water) without serum, negative control serum or positive control serum. A twofold dilution was done with the positive control. Sera were prediluted 1/10 or 1/25. At 48 h p. i., cells were fixed with 4% paraformaldehyde (PFA), 3.5% PFA or 80% acetone and analyzed by icELISA. Bars depict the mean values ± SD. Dots show the values of the individual measurements. Two-fold replicates of samples were determined. (TIF) [file pntd.0010425.s002.tif]

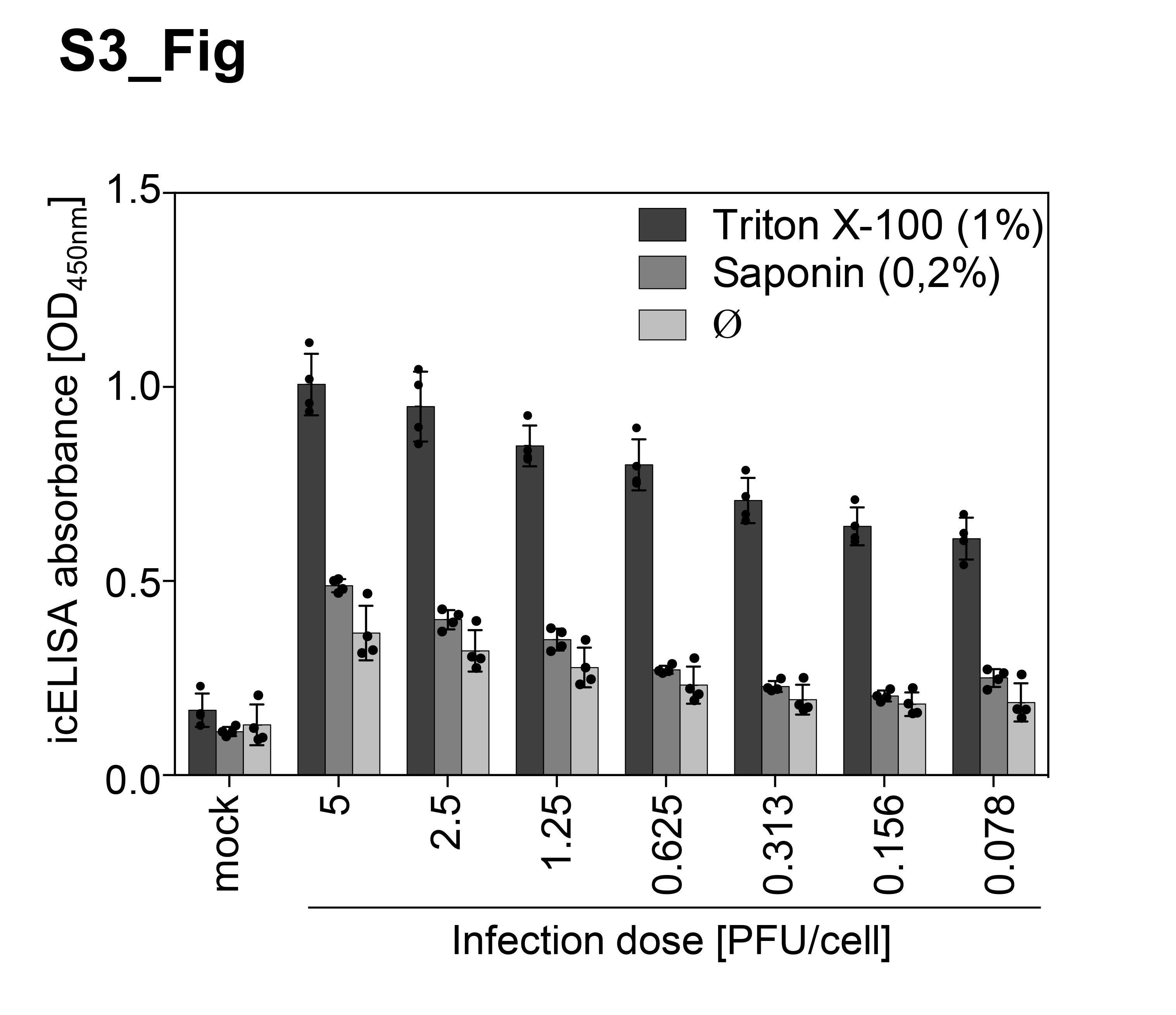

Supplement: S3 Fig — BHK-21 cells were infected with Rabies lyssavirus using the indicated virus dose. At 22 h p. i., cells were fixed, permeabilized with 1% Triton-X-100, 0.2% Saponin, or without detergents and analyzed by icELISA. Bars depict the mean values ± SD. Dots show the values of the individual measurements. Cell were fixed after 22 h p. i. because the experiment was conducted prior to the experiments that assessed the influence of different infections periods. Four-fold replicates of samples were determined. (TIF) [file pntd.0010425.s003.tif]

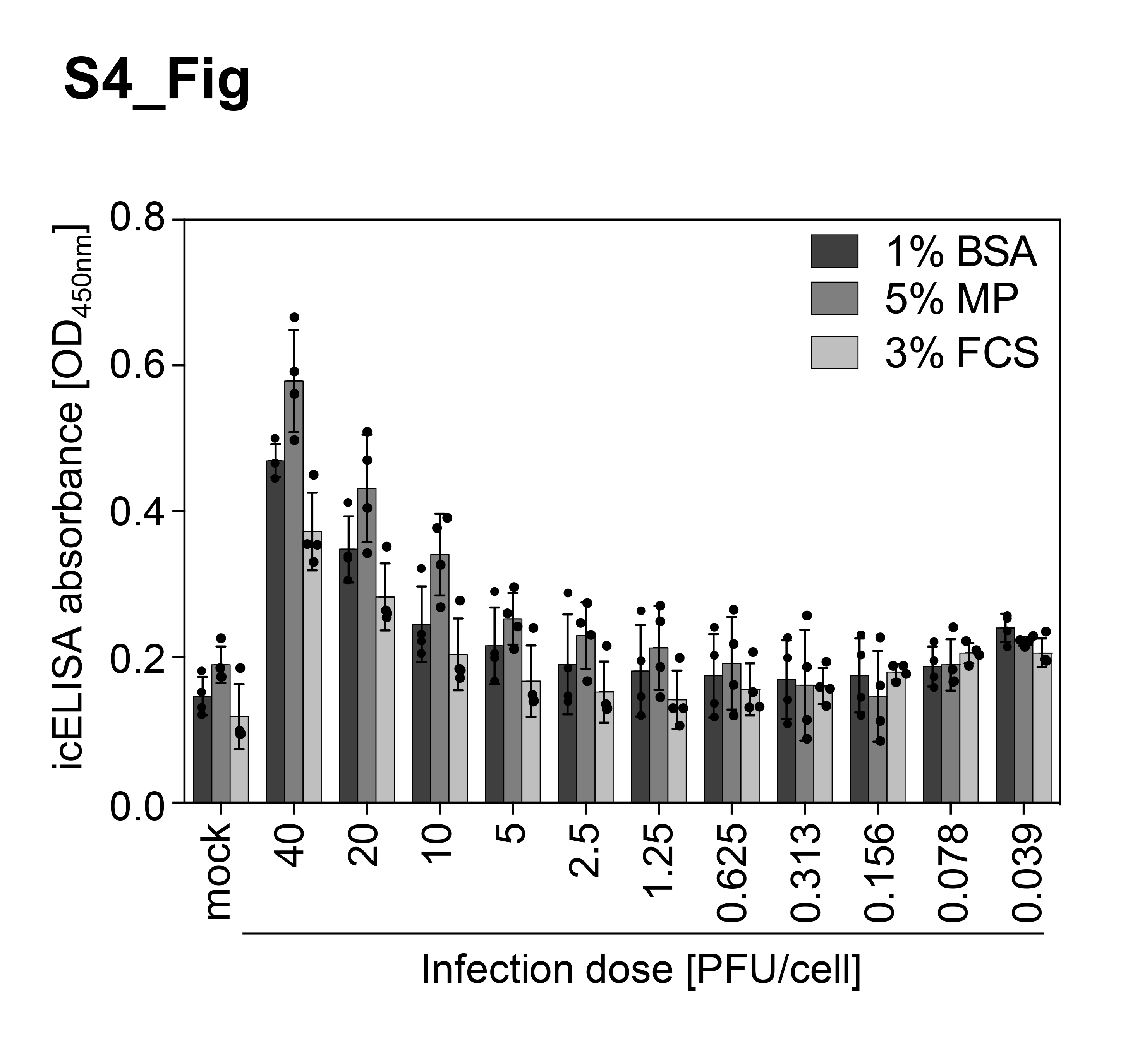

Supplement: S4 Fig — BHK-21 cells were infected with Rabies lyssavirus using the indicated virus dose. At 22 h p. i., cells were fixed, blocked with 1% Bovine serum albumin (BSA), 5% milk powder (MP), or 3% fetal calf serum (FCS) and analyzed by icELISA. Bars depict the mean values ± SD. Dots show the values of the individual measurements. Cell were fixed after 22 h p. i. because the experiment was conducted prior to the experiments that assessed the influence of different infections periods. Four-fold replicates of samples were determined. (TIF) [file pntd.0010425.s004.tif]

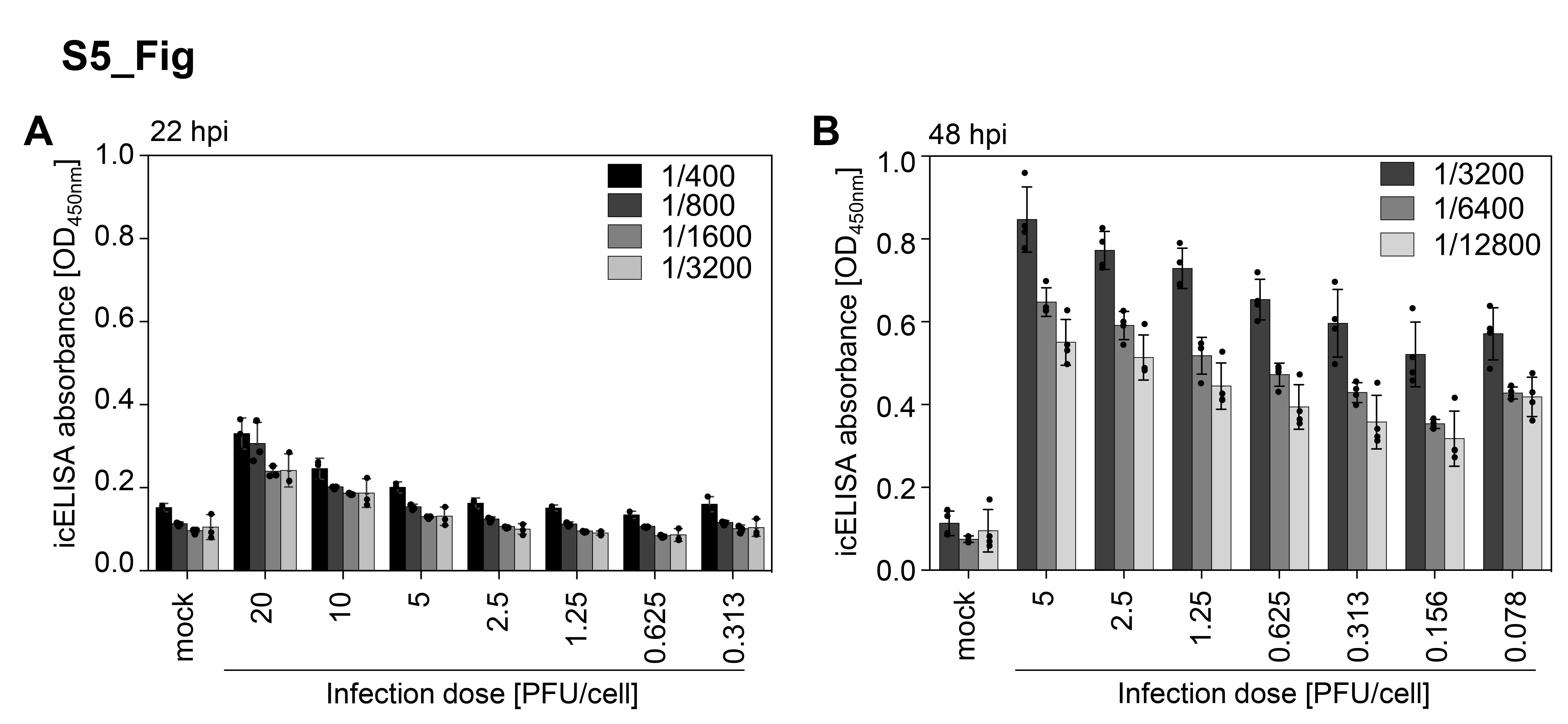

Supplement: S5 Fig — BHK-21 cells were infected with Rabies lyssavirus using the indicated virus dose. (A) At 22 h p. i., cells were fixed and analyzed by icELISA using indicated dilutions of Anti-Rabies Monoclonal Globulin. Three-fold replicates of samples were determined. (B) At 48 h p. i., cells were fixed and analyzed by icELISA using indicated dilutions of Anti-Rabies Monoclonal Globulin. Four-fold replicates of samples were determined. Bars depict the mean values ± SD. Dots show the values of the individual measurements. (TIF) [file pntd.0010425.s005.tif]

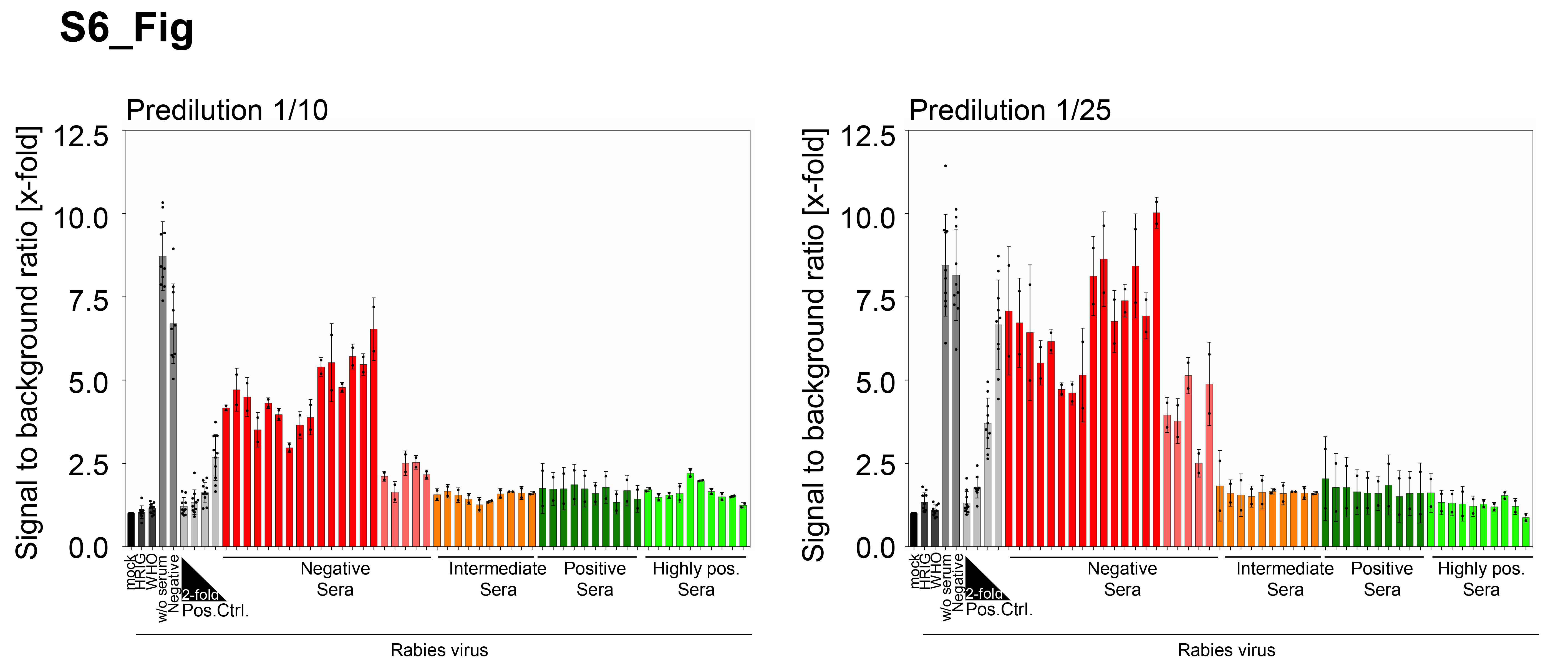

Supplement: S6 Fig — HRIG, WHO SRIG, negative control serum, twofold dilution of positive control serum, 20 seronegative, 10 intermediate, 10 positive, and 10 strongly positive serum samples with known RFFIT titers were analyzed by icNT. Serum samples were prediluted 1/10 or 1/25. The different graphs each represent one predilution. Each measurement was performed in duplicate. (TIF) [file pntd.0010425.s006.tif]
