# Supplementary material for: Establishment and clinical validation of an in-cell-ELISA-based assay for the rapid quantification of Rabies lyssavirus neutralizing antibodies
Source: PLoS Negl Trop Dis. 2022 May 10;16(5):e0010425. doi: 10.1371/journal.pntd.0010425 (PMC9159627; doi:10.1371/journal.pntd.0010425)
Supplement: S1 Text — A detailed laboratory protocol for the icNT and the icELISA is provided. (DOCX) [file pntd.0010425.s007.docx]

RABV icNT (steps 1 to 12) and icELISA (steps 5 to 12)

Materials:

- BHK-21 cells
- MEM Eagle with Earle's Balanced Salt Solution (EBSS), L-Glutamine (292 mg/L), and 2·2 g/L NaHCO_3_ supplemented with 10% (v/v) FCS, 1% ZellShield
- Virus stock RABV
- Primary and secondary antibody (e.g., Anti-Rabies Monoclonal Globulin, α-mouse IgG POD-coupled)
- Paraformaldehyde (PFA)
- 1x PBS (PBS), 2x PBS
- Triton-X-100
- Tween-20
- Fetal Calf Serum (FCS)
- Tetramethylbenzidine (TMB)
- 0·5 M HCl
- Distilled or deoinized water

Equipment:

- 96-well microplate
- 37°C CO_2_ incubator
- Multichannel pipette
- Microplate reader

Notes:

- The wells of the microplate should not be allowed to dry at any point during the assay procedure.
- It is recommended to use a plate shaker during the incubation steps of the icELISA.

| **REAGENT** | **REAGENT PREPATION INSTRUCTION** | **WHEN TO PREPARE?** |
| --- | --- | --- |
| Wash Buffer | 500µl Tween-20 + 1 liter PBS | At start of experiment |
| 8% (w/v) Paraformaldehyde Solution | 1. Dissolve a PBS tablet in 500 ml distilled or deionized water (= 2xPBS) 2. Add 80 gr PFA 3. Use 4 M NaOH to dissolve the PFA 4. Use HCl for pH adjustment (pH 7·4) 5. Fill up with distilled or deionized water (1 liter) | At start of experiment  (store in aliquots  at -20°C) |
| Permeabilization Buffer | For one 96-well microplate:  Dilute 250µl Triton-X-100 in 24·75 ml PBS. Mix well by vortexing. | Prepare immediately before use |
| Blocking Solution | 3% (v/v) FCS in PBS | Prepare immediately before use |
| Incubation Buffer | 1% (v/v) FCS in PBS | Prepare immediately before use |

1. Prepare the serum dilutions within a 96-well microplate:
   1. Add 100µl medium per well to row A-C and E-G.
   2. Dilute the control serum and the serum samples to be tested 1/10 and add 167µl per well to row D and H.
   3. Perform 2·5-fold serial dilutions by adding 67µl to the well above.
   4. Discard the excess amount of the last dilution.
   5. Include the control samples on every plate.
2. Prepare the virus solution (5·5 ml per 96-well microplate).
   1. Calculate the required amount of total virus.
   2. Prepare the virus solution by diluting the required amount of virus stock in medium.
   3. Include the mock and virus controls on every plate.
3. Neutralization: Incubation of virus with serum.
   1. Add 50µl virus solution per well to the prepared serum dilutions.
   2. Incubate for 70 ± 5 min at 37°C.
4. Infection.
   1. Prepare BHK-21 cell suspension (~2·5*10^4^/well).
   2. Add 50µl of BHK-21 cell suspension per well.
5. Fix cells to microplate.
   1. At 48 h p.i., add 150µl of 8% paraformaldehyde solution to the wells.
   2. Incubate for 15 min at room temperature.
6. Replace the lids with new ones.
7. Gently aspirate the fixing solution from the microplate. Wash the microplate 3 times with 300µl PBS per well. Add 200µl PBS to the wells. The microplate (sealed with parafilm) can now be stored at 4°C for several days.
8. Permeabilize cells.
   1. Prepare permeabilization buffer (for one microplate: dilute 250µl Triton-X-100 in 24·75 ml PBS).
   2. Aspirate PBS and add 200µl of freshly prepared permeabilization buffer to each well.
   3. Incubate for 30 min.
9. Blocking.
   1. Aspirate permeabilization buffer and add 200µl of blocking solution (3% [v/v] FCS in PBS) to each well.
   2. Incubate for 2 h.
10. Incubation with primary antibody.
    1. Prepare primary antibody (Anti-Rabies Monoclonal Globulin, 1:4200) by diluting stock antibody in the required volume of incubation buffer (1% [v/v] FCS in PBS).
    2. Aspirate blocking solution and add 50µl of antibody solution to each well.
    3. Incubate for 35-40 minutes at 37°C, for 2 h at room temperature, or overnight at 4°C (sealed with parafilm).
11. Incubation with secondary antibody.
    1. Prepare antibody solution (goat anti-mouse (GAM) antibody, 1:2000) by diluting stock antibody in the required volume of incubation buffer (1% [v/v] FCS in PBS).
    2. Aspirate primary antibody solution. Wash the microplate 3 times with 250µl wash buffer per well.
    3. Aspirate the wash buffer and add 50µl of secondary antibody solution to each well.
    4. Incubate for 2 h at room temperature.
    5. Aspirate secondary antibody solution. Wash 4 times with 250µl wash buffer per well.
12. Signal measurement.
    1. Prepare the required amount of TMB and 0·5 M HCl.
    2. Aspirate the last wash. Make sure that you have completely removed the liquid.
    3. Add 100µl TMB (blue color development).
    4. Stop the reaction with 100µl of 0·5 M HCl (by the time the uninfected wells [mock] are starting to turn blue as well).
    5. Record data at 450 nm absorbance, 620 nm reference using a microplate reader.
